# *Ascophyllum nodosum* Biostimulant Improves the Growth of *Zea mays* Grown Under Phosphorus Impoverished Conditions

**DOI:** 10.3389/fpls.2020.601843

**Published:** 2021-01-08

**Authors:** Pushp Sheel Shukla, Balakrishnan Prithiviraj

**Affiliations:** Marine Bio-Products Research Laboratory, Department of Plant, Food and Environmental Sciences, Faculty of Agriculture, Dalhousie University, Truro, NS, Canada

**Keywords:** *Ascophyllum nodosum* extract, P-limited condition, chemical fertilizer, gene expression, *Zea mays*

## Abstract

Phosphorous is one of the major limiting factors determining plant growth. Current agricultural practices mainly rely on the use of chemical fertilizers posing threat to the ecosystem. In this study, the application of an *Ascophyllum nodosum* extract (ANE) in phosphorous (P)-limited conditions improved the fresh and dry weight of shoots and roots of *Zea mays*. ANE-treated *Z. mays* grown under P-limited conditions showed a higher P content than the control. ANE activated simultaneous responses, at multiple levels, in *Z. mays* grown under P-limited conditions as seen from the regulation of gene expression at the whole-plant level to specific biochemical responses on a subcellular level. ANE-supplemented *Z. mays* grown under P-limited conditions also showed reduced electrolyte leakage and lipid peroxidation by an improved membrane stability. ANE treatment reduced P-limitation-induced oxidative damage in *Z. mays* by reducing H_2_O_2_ and ${\text{O}}_{2}^{-}$ accumulation. Furthermore, ANE also induced the accumulation of the total contents of soluble sugars, amino acids, phenolics, and flavonoids. Gene expression analysis suggested that ANE differentially modulated the expression of P-starvation responsive genes involved in metabolic, signal transduction, and developmental pathways in *Z. mays*. ANE also modulated the expression of genes involved in sugar, lipid, and secondary metabolism. Thus, this study illustrated the role of ANE in improving the productivity of *Z. mays*, an important crop, in P-limited conditions. Furthermore, it sets the framework to increase agricultural productivity in nutrient deficient soils using a sustainable, eco-friendly strategy.

## Introduction

Phosphorous (P) is an important macronutrient for plants and constitutes about 0.2% of dry weight (DW) (Schachtman et al., 2002). Phosphorous is a key constituent of cellular biomolecules, such as ATP, NADP, nucleic acids, phospholipids, sugar phosphates, and enzymes (Hernández and Munné-Bosch, 2015; Achary et al., 2017). It plays an important role in nucleic acid and protein synthesis, membrane integrity, photosynthesis, respiration, energy metabolism, hormone regulation, stress tolerance, and disease resistance (Sun et al., 2018). Adequate P availability is necessary for normal plant growth (Vance et al., 2003). Globally, 5.7 billion ha of all the available agricultural land is defined as being deficient in phosphorous due to soil degradation and is a major limiting factor of agricultural productivity (Roch et al., 2019).

Chemical fertilizers are used to enhance the availability of P to plants from the soil (Veneklaas et al., 2012; Heuer et al., 2017). However, the excessive use of chemical fertilizers poses risks to the environment. In addition, the raw material (i.e., rock phosphate) used to manufacture P fertilizer is a finite, natural source, and thus the costs of P fertilizers can only increase (Vance et al., 2003). Attempts have been made to genetically engineer selected plants with a higher P-use efficiency through transformation of genes involved in P acquisition, transportation, and signal transduction (Hammond, 2003; Chiou, 2005). However, P uptake has been found to be a multi-genic trait; limited success had been achieved in generating P-efficient crops (Shenoy and Kalagudi, 2005; George and Richardson, 2008). In the current scenario, there is a need to develop a more sustainable strategy to enhance P-use efficiency in agricultural systems.

Seaweed-derived biostimulants are new class of agricultural input that are being widely researched for the improvement of various nutrient-use efficiencies in plants (Khan et al., 2009; Jannin et al., 2013). *Ascophyllum nodosum* is a brown, intertidal seaweed, common to the Northern Hemisphere and has been extensively investigated as a source of various commercial biostimulants with the specific aims of improving plant growth and productivity by increasing nutrient availability and uptake (Jannin et al., 2013; Shukla et al., 2019; Pereira et al., 2020). AZAL5 is a commercial extract of *A. nodosum* and plays a vital role in nitrogen (N), carbon (C), and sulfur (S) metabolism in *Brassica napus* (Jannin et al., 2013). In the same study, micro-array analysis of *B. napus* treated with AZAL5 showed differential regulation of genes involved in nitrate assimilation and amino acid metabolism. Another commercial extract of *A. nodosum* extract (ANE) improved salinity tolerance in *Arabidopsis* by improving P uptake in salt-stressed plants by regulating the expression of miRNA involved in P homeostasis (Shukla et al., 2018). Applications of ANE also enhanced the macro- and micro-nutrient contents of tomato fruits grown under salinized conditions (Di Stasio et al., 2018). The aim of this study was to elucidate the mechanisms of action of ANE in improving the growth of *Zea mays* grown under phosphorous (P)-limited conditions.

## Materials and Methods

### Treatment of *Z. mays* With Experimentally Induced P-Limiting Conditions

*Z. mays* seeds (cv. Bilicious) were purchased from Halifax Seed (Halifax, NS, Canada). ANE, commercially available as Acadian Marine Plant Extract (Acadian Seaplants Ltd., Dartmouth, NS, Canada), was used as the seaweed biostimulant. The chemical composition of the extract was described by Rayirath et al. (2009). The P content in the 0.01% of ANE was measured using inductively coupled plasma optical emission spectrometer (ICP-OES) at the Analytical and Dairy Lab, Harlow Institute, Nova Scotia, Canada.

Seeds were sterilized using 2.5% Clorox® and were kept on filter paper moistened with water in a petri dish for germination in the dark. Seven-day-old, uniformly germinated, seedlings were used for further experiments. The experiment was designed to assess the effects of 0.01% ANE on the growth of 7-day-old *Z. mays* seedlings grown under P-limited conditions for 14 days. This concentration of ANE was selected based on the results reported in previous studies (Shukla et al., 2018; Bajpai et al., 2019; Jithesh et al., 2019). In addition to the control (1/2 MS (Murashige and Skoog media), C), the other three treatments were 1/2 MS + 0.01% ANE (T_1_), 1/2 MS-P (T_2_), and 1/2 MS-P + 0.01% ANE (T_3_). The MS medium without P (MSP19) was procured from Caisson Labs, USA. It was estimated that 0.01% ANE contained 5.0 μM P, which was added to T_2_ in the form of H_3_PO_4_ (Rickard, 2000). The plants treated with different treatments were grown for 14 days in the growth chamber maintained at 24°C with a photoperiod (100 μmol photons/m^2^/s) of 16/8 h (day/night). Increase in total root length and leaf area of plants was recorded at 14 days after the imposition of P-limited conditions by using ImageJ software. After 14 days under different conditions, fresh weights of shoots and roots were recorded. DW was recorded after drying in an oven at 70°C for 72 h. The data for the morphological parameters were generated using six plants for each experimental condition, and the experiment was triplicated.

### Mineral Element Analysis

The nitrogen content and mineral element analysis were done at the Analytical and Dairy Lab, Harlow Institute, Nova Scotia, Canada. The plants grown under different treatments for 14 days were dried at 72°C for 72 h. The nitrogen content in the plant tissue was determined by combustion analysis method using Leco CN828 Carbon Nitrogen Determinator. The mineral elements were measured from the ash dried plant samples with an ICP-OES.

### Determination of Pigments

The effect of ANE on chlorophyll a and b and the carotenoid content of *Z. mays* grown under different treatments for 14 days were determined according to the protocol described by Lichtenthaler (1987). Briefly, 1 g of six individual plant samples was instantaneously ground using a mortar and pestle in 5 ml of cold methanol. Following extraction, the ground mixture was centrifuged at 10,000 rpm at 4°C for 10 min, and the pellet was re-extracted with 10 ml of cold methanol until all color was removed. Two extracts were combined, and total volume was made up to 15 ml. Absorbance was measured at 470, 652.4, and 665.2 nm using a UV–VIS spectrophotometer (Biotek, USA). The chlorophyll contents were calculated according to Lichtenthaler (1987).

Chl_a_ = 16.72 A_665.2_ – 9.16 A_652.4_

Chl_b_ = 34.09 A_652.4_ – 15.28 A_665.2_

Carotenoids = (1,000 A_470_ – 1.63 Chl_a_ – 104.96 Chl_b_) / 221

### Determination of Anthocyanin Content

The effect of ANE on the anthocyanin content of *Z. mays* grown under different treatments for 14 days was evaluated using the protocol published by Burgos et al. (2013). One gram of plant sample was extracted with 10 ml of methanol and acidified with 1% HCl. The mixture was centrifuged at 5,000 rpm for 10 min at 4°C, and the pellet was re-extracted. Different fractions were combined, and the final extract volume was made up to 25 ml. Absorbance at 545 nm was recorded using a UV–VIS spectrophotometer. The anthocyanin content was calculated using the molar extinction coefficient and molecular weight of malvidin-3-p-coumaroyl-glucoside (i.e., 545 nm, 3.02 × 104 L/mol/cm, 718.5 g/mol).

### Determination of Electrolyte Leakage and Membrane Stability Index

Electrolyte leakage was determined as described by Shukla et al. (2012). The youngest, fully expanded leaves were collected from plants grown under different treatments for 14 days. The leaves were thoroughly cleaned with de-ionized water to remove surface-adhered electrolyte. The samples were placed in a closed, glass tube containing 10 ml of de-ionized water. After a 24-h incubation at 25°C on a rotary shaker, electrical conductivity (L_t_) was measured (Hanna Instruments, Canada). The samples were autoclaved at 120°C for 20 min and cooled to room temperature to determine the final electrical conductivity (L_0_). Electrolyte leakage was calculated according to Lutts et al. (1996).

$$\begin{array}{l} \text{Electrolyte leakage }\left(\%\right)=\left(\frac{{\text{L}}_{\text{t}}}{{\text{L}}_{0}}\right)\times 100 \end{array}$$

A membrane stability index (MSI) was determined according to the protocol published by Yadav et al. (2012). In this method the youngest, fully expanded leaves were collected from the plants grown under different treatments for 14 days and placed into 10 ml of de-ionized water in two sets, as triplicates. The electrical conductivity (C_1_) of one set of samples was measured after heating at 40°C for 30 min in a water bath. The second set was boiled at 100°C for 10 min; electrical conductivity (C_2_) was measured after cooling to room temperature. MSI was calculated using the following formula:

$$\begin{array}{l} \text{MSI}=\left[1-\left(\frac{{\text{C}}_{1}}{{\text{C}}_{2}}\right)\right]\times \;100. \end{array}$$

### Determination of Lipid Peroxidation

Lipid peroxidation was estimated by determining the concentration of malondialdehyde (MDA) in the leaves of the plants grown under different treatments for 14 days, after Hodges et al. (1999). Leaf material (0.5 g) was homogenized in 15 ml of 80% ethanol (EtOH), followed by centrifugation at 5,000 rpm at 4°C for 10 min. The supernatant (100 μl) and distilled water (900 μl) were mixed in a test tube with 1 ml of either (i) –TBA (thiobarbituric acid) solution including 20% (w/v) trichloroacetic acid (TCA) and 0.01% (w/v) butylated hydroxytoluene (BHT) or (ii) +TBA solution containing 0.65% (w/v) TBA with 20% (w/v) TCA and 0.01% (w/v) BHT. The mixture was vortexed, heated at 95°C in a dry bath for 25 min, cooled, and centrifuged at 5,000 rpm for 10 min. Absorbance was measured at 440, 532, and 600 nm. MDA equivalents were calculated using the following formula:

(1) [(Abs_532+TBA_) – (Abs_600+TBA_) – (Abs_532−TBA_ – Abs_600−TBA_)] = A,

(2) [(Abs_440+TBA_ – Abs_600+TBA_) 0.0571] = B,

(3) MDA equivalents (nmol/ml) = 10^6^ [(A – B) / 157,000].

### *In vivo* Localization and Quantification of ${\text{O}}_{2}^{-}$ and H_2_O_2_

*In vivo* localization and quantification of ${\text{O}}_{2}^{-}$ and H_2_O_2_ were performed by histochemical staining with nitro-blue tetrazolium (NBT) and 3,3′-diaminobenzidine (DAB), as described by Yadav et al. (2012). To stain ${\text{O}}_{2}^{-}$ in the leaves of the *Z. mays* grown under treatments C, T_1_, T_2_, and T_3_, leaf samples were immersed in 1 mg ml^−1^ of NBT in 10 mM phosphate buffer. After a 12-h incubation at room temperature, blue colored spots were observed, indicative of ${\text{O}}_{2}^{-}$ formation. Similarly, H_2_O_2_ was stained by incubating leaf samples in 1 mg ml^−1^ of DAB for 24 h. After incubation, brown colored spots were formed as a result of the reaction between DAB and H_2_O_2_.

${\text{O}}_{2}^{-}$ content was estimated in leaves of *Z. mays*, as described by Liu and Pang (2010). Leaf tissue was homogenized in 10 ml of 65 mM potassium phosphate buffer (pH 7.8) and centrifuged at 5,000 rpm for 10 min. The reaction mixture consisted of 0.9 ml of 65 mM phosphate buffer (pH 7.8) and 0.1 ml of 10 mM hydroxylamine hydrochloride, and 1 ml of the extract was incubated at 25°C for 20 min. Then, 17 mM sulfanilamide and 7 mM α-naphthylamine were added to the extract, the mixture was further incubated at 25°C for 20 min, and absorbance was read at 530 nm using a UV–VIS spectrophotometer. A standard curve (10–200 nmol) was prepared with NaNO_2_ to calculate the production rate of ${\text{O}}_{2}^{-}$. The H_2_O_2_ content in leaf samples was measured as described by Yadav et al. (2012). Leaf tissue was extracted with cold acetone. Two milliliters of the extract was mixed with 0.5 ml of 0.1% titanium dioxide in 20% (v*/*v) H_2_SO_4_, and the mixture was centrifuged at 6,000 rpm for 15 min. The intensity of yellow color of the supernatant was measured at 415 nm using a UV–VIS spectrophotometer.

### Determination of Total Soluble Sugars and Total Amino Acids

The effect of ANE treatments on the total soluble sugars and amino acids of *Z. mays* was determined according to the protocol described by Irigoyen et al. (1992) and Shukla et al. (2012), respectively. Leaf samples (100 mg) from all the treatments were frozen in liquid nitrogen and homogenized with mortar and pestle. The fine frozen powder was immediately suspended in 5 ml of 80% EtOH. The mixture was centrifuged at 5,000 rpm for 10 min at 4°C, and the pellet was re-extracted. Fractions were combined, and the final volume was made up to 15 ml. The total soluble sugars were analyzed by reacting 100 μl of alcoholic extract with 3 ml of freshly prepared anthrone reagent (150 mg anthrone in 100 ml of 72% (v/v) H_2_SO_4_). The mixture was placed in a boiling water bath for 10 min. After the mixture was cooled to room temperature, absorbance at 620 nm was measured. The content of total amino acids was determined by treating 1 ml of alcoholic extract with 1 ml of 0.2 M citrate buffer (pH 5.0), 1 ml of 80% EtOH, and 1 ml of ninhydrin (1%), followed by incubation at 95°C for 15 min. The samples were cooled, and the absorbance at 570 nm was measured.

### Determination of Total Phenolics and Flavonoids Contents

For the quantification of secondary metabolites, *Z. mays* leaves were harvested from different treatments after 14 days. The samples were frozen in liquid nitrogen and homogenized with mortar and pestle. The fine frozen powder was immediately suspended in 70% methanol and centrifuged at 10,000 rpm for 10 min at 4°C. The total phenolics content was determined using Folin-Ciocalteu assay as described by Hodges and Lester (2006). One milliliter of extract was treated with 0.2 N Folin-Ciocalteu phenol reagent and vortexed. After incubation of the mixture for 5 min at room temperature, 1 ml of 7% Na_2_CO_3_ solution was added to the mixture. The mixture was incubated for 90 min at room temperature. After incubation, the absorbance against the reagent blank was determined at 550 nm. Total phenolics content was expressed as gallic acid equivalents (mg/g FW).

Total flavonoids content was determined according to Hichem et al. (2009). Briefly, 1 ml of plant extract was added to 4 ml of distilled water. The diluted samples were treated with 0.3 ml of 5% sodium nitrite (NaNO_2_). After 5 min of incubation at room temperature, 0.3 ml of 10% aluminum chloride was added. Then, after 5 more minutes, 2 ml of 1 M sodium hydroxide (NaOH) was added to the mixture, and the final volume was made up to 10 ml. The solution was vortexed, and absorbance was measured against the reagent blank at 510 nm. The total flavonoids content was expressed as mg quercetin equivalents (QE).

### Gene Expression Analysis

To elucidate the role of ANE treatments in improving plant growth under P-limited conditions, real-time expression of genes involved in P homeostasis, carbohydrate metabolism, lipid metabolism, and secondary metabolism were performed. For gene expression analysis, experiments were conducted in a hydroponic system. Seven-day-old seedlings were inserted into a polystyrene disc floated on 1/2 MS (C), 1/2 MS + 0.01% ANE (T_1_), 1/2 MS-P + 5 μM H_3_PO_4_ (T_2_), and 1/2 MS-P + 0.01% ANE (T_3_) in 300 ml Phyta jars. Leaf and root samples were harvested after 2 and 7 days of treatment. Total RNA was extracted using the RNAeasy kit (Qiagen, Germany) following the manufacturer's protocol. The quantity and purity of the total RNA were analyzed using a NanoDrop 2000 spectrophotometer (Thermo Scientific, USA). An amount of 2.5 μg RNA from each sample was treated with DNase I (Promega, USA), and then the synthesis of cDNA was performed using the Revert Aid cDNA reverse transcription kit (Thermo Scientific, USA). Real-time quantitative PCR (qPCR) was performed using the StepOnePlus Real-Time PCR system (Applied Biosystems). Actin and β-tubulin were used as reference genes. A list of the different primers used in this study is presented in Supplementary Table 1. The specificity of PCR amplification was checked at the end of the PCR cycles by melt-curve analysis. Each biological sample had three technical replicates, and the relative-fold expression was determined using the Livak (2^−ΔΔCt^) method (Livak and Schmittgen, 2001).

### Statistical Analysis

Each experiment was carried out in triplicate, each experimental unit had six plants, and for each response variable, the average of the 18 values was used for statistical analysis. The results were presented as mean ± standard error. Data were analyzed using ANOVA, with a *p* ≤ 0.01 using the “Proc. mixed procedure,” of the SAS Institute, Inc. software version 9.3 (SAS Institute, Inc., Cary, NC, USA). When significant effects of treatments were found, multiple means comparison was carried out using Tukey's analysis with a 95% confidence interval. The significantly different mean values were represented by different alphabets.

## Results

### ANE Improved Growth of *Z. mays* Grown Under P-Limited Conditions by Regulating Nutrient Homeostasis

**Figure 1:**
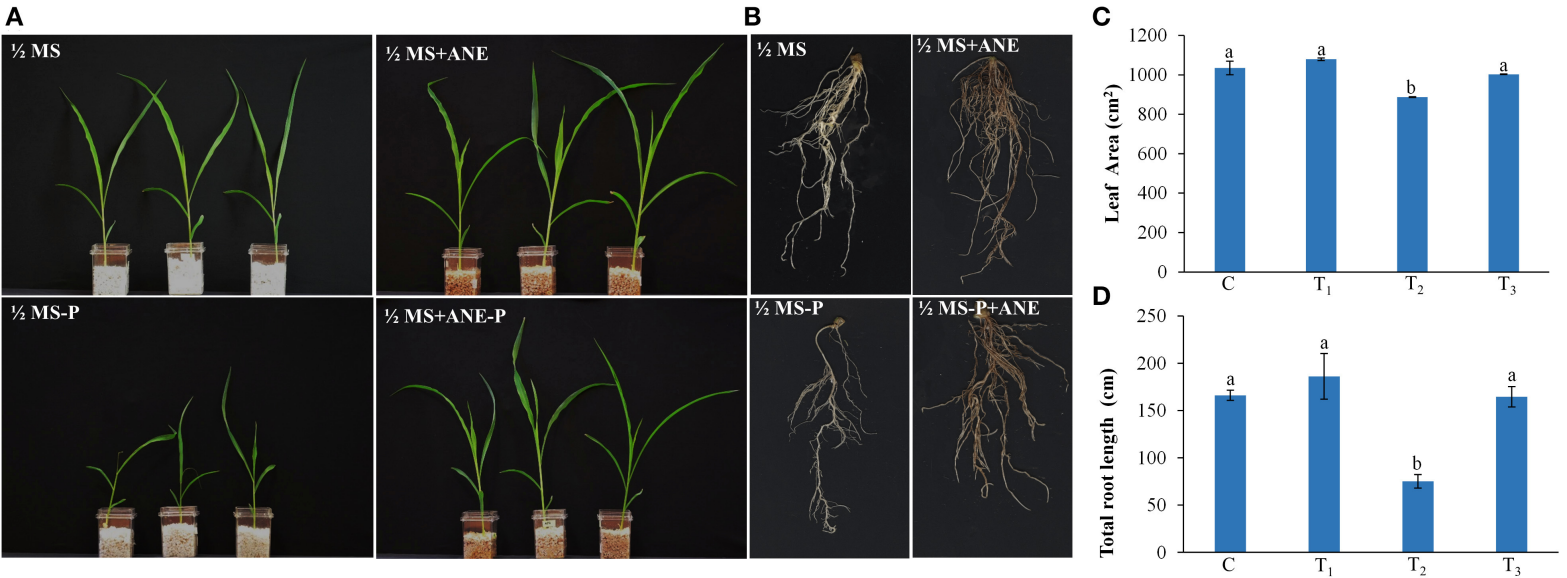
*Ascophyllum nodosum* extract (ANE) improves the growth of *Z. mays* grown under P-limiting conditions. Effect of ANE on **(A)** shoot, **(B)** root growth, **(C)** leaf area, and **(D)** total root length of *Z. mays* under the P-limiting conditions. Seven-day-old *Z. mays* seedlings were grown in perlite supplemented with 1/2 MS and 1/2 MS-P for 14 days. C (control): plant grown in perlite supplemented with 1/2 MS, T_1_: plant grown in perlite supplemented with 1/2 MS + ANE, T_2_: plant grown in perlite supplemented with 1/2 MS-P, T_3_: plant grown in perlite supplemented with 1/2 MS-P + ANE. The values were presented as mean ± SE, and means represented by the same letters are not significantly different at *p* ≤ 0.01. Each experiment was carried out in triplicate, and each experimental unit had six plants (*n* = 18).

**Figure 2:**
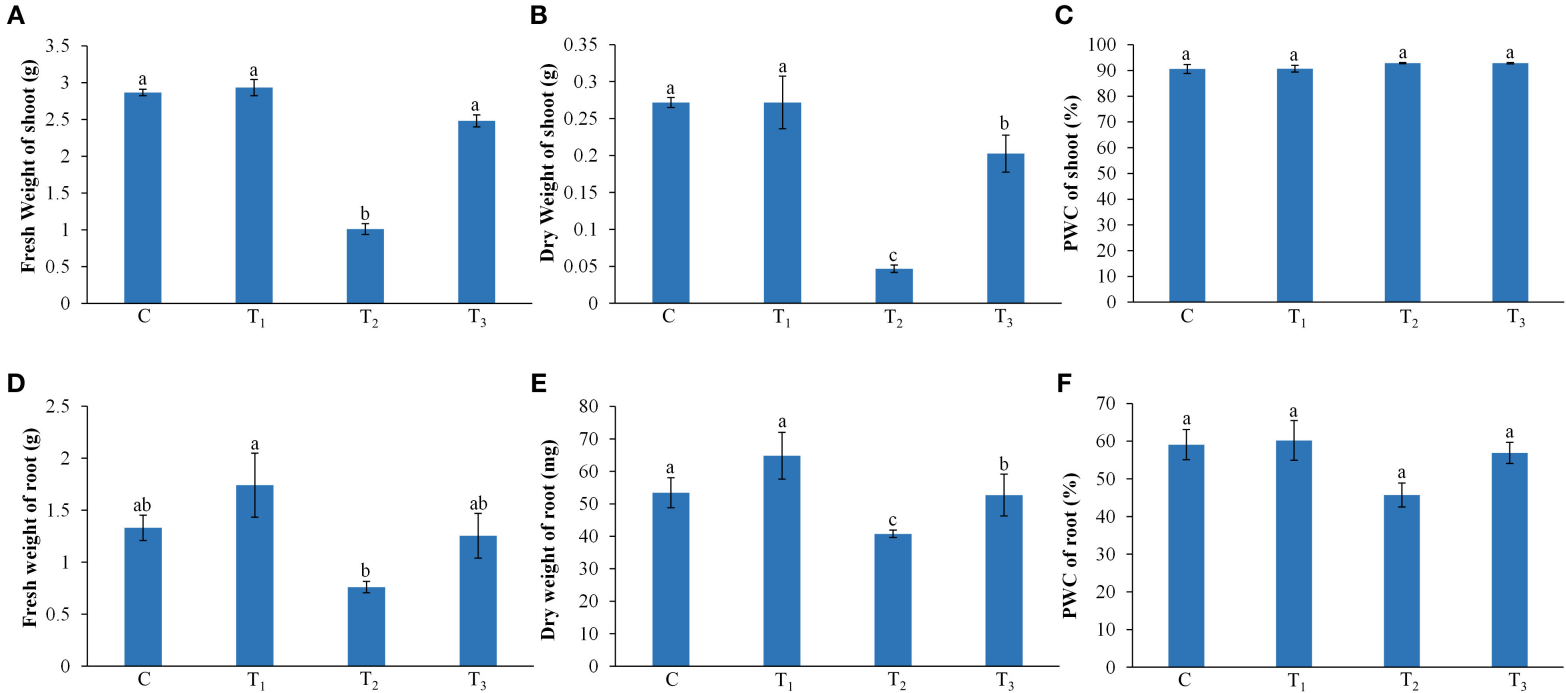
Effect of ANE on fresh weight, dry weight, and percentage water content (PWC) of shoot **(A–C)** and root **(D–F)**, respectively, of *Z. mays* grown under P-limiting conditions. C (control): plant grown in perlite supplemented with 1/2 MS, T_1_: plant grown in perlite supplemented with 1/2 MS + ANE, T_2_: plant grown in perlite supplemented with 1/2 MS-P, T_3_: plant grown in perlite supplemented with 1/2 MS-P + ANE. The values were presented as mean ± SE, and means represented by the same letters are not significantly different at *p* ≤ 0.01. Each experiment was carried out in triplicate, and each experimental unit had six plants (*n* = 18).

Under low P conditions, the shoot and root growth of the *Z. mays* were significantly reduced, as compared with the control (Figure 1). The addition of ANE in the growth media improved the growth of the plant under P-limited conditions (Figure 1). Leaf area and total root length of the ANE-supplemented plants were significantly higher (*p* ≤ 0.01) in P-limited conditions than those of the control (Figures 1C,D). ANE-supplemented *Z. mays* plants grown under P-limited conditions showed higher fresh and DWs of shoots and roots, but not their percentage water content (PWC), than the control (Figure 2). The dry biomass of the shoot and root of ANE-supplemented *Z. mays* grown under P-limited conditions was found to be 4.33 and 1.29 times, respectively, higher than the plants grown in P-limited conditions alone (Figures 2B,E).

Mineral analysis was carried out on the leaves of the *Z. mays* plants grown under different treatments [1/2 MS (control, C), 1/2 MS + 0.01% ANE (T_1_), 1/2 MS-P (T_2_), and 1/2 MS-P + 0.01% ANE (T_3_)] (Table 1). No significant differences were observed in the nitrogen (N), phosphorus (P), potassium (K), calcium (Ca), magnesium (Mg), sodium (Na), zinc (Zn), and copper (Cu) contents of those plants grown in 1/2 MS and 1/2 MS + 0.01% ANE. P-limited conditions did not appear to have any influence on the K, Ca, and Na contents of the *Z. mays*. However, P-limited conditions significantly (*p* ≤ 0.01) reduced the N content in the plants, whereas ANE supplementation in the P-limited media increased N content by 18.3% in those plants. The application of ANE in the P-limited media significantly increased the P content, suggesting that ANE restored growth of those plants, in P-limited conditions, by regulating P homeostasis. Those plants grown in the presence of 1/2 MS-P had a reduction in Mg content of 30.2% and Zn by 26.25%, as compared with the control, whereas ANE treatments showed significantly less reduction in Mg content by 11.9% and Zn content by 15.3% under P-limited conditions, as compared with the control (Table 1).

**Table 1:** Mineral content in the leaves of *Z. mays* grown in the perlite supplemented with 1/2 MS (C), 1/2 MS + ANE (T_1_), 1/2 MS-P (T_2_), and 1/2 MS-P + ANE (T_3_).

	**N (mg/g DW)**	**P (mg/g DW)**	**K (mg/g DW)**	**Ca (mg/g DW)**	**Mg (mg/g DW)**	**Na (mg/g DW)**	**Fe (μg/g DW)**	**B (μg/g DW)**	**Mn (μg/g DW)**	**Zn (μg/g DW)**	**Cu (μg/g DW)**
C	6.19 ± 0.15^a^	6.19 ± 1.27^a^	5.27 ± 0.23^a^	0.37 ± 0.01^a^	0.21 ± 0.011^a^	0.52 ± 0.018^a^	117.26 ± 0.14^d^	27.02 ± 0.11^d^	109.24 ± 0.35^d^	116.94 ± 0.00^a^	8.04 ± 0.02^c^
T_1_	5.35 ± 0.11^ab^	5.95 ± 0.94^a^	5.42 ± 0.38^a^	0.30 ± 0.00^a^	0.19 ± 0.006^a^	0.40 ± 0.017^ab^	137.37 ± 5.13^c^	38.38 ± 0.13^c^	117.61 ± 0.35^c^	113.88 ± 6.95^a^	8.30 ± 0.38^c^
T_2_	4.76 ± 0.53^b^	3.76 ± 0.21^b^	4.20 ± 0.24^b^	0.27 ± 0.02^b^	0.14 ± 0.011^b^	0.29 ± 0.015^bc^	236.43 ± 6.75^a^	51.49 ± 0.89^b^	124.88 ± 1.46^b^	85.81 ± 2.43^c^	9.59 ± 0.03^b^
T_3_	5.63 ± 0.26^ab^	5.53 ± 0.68^a^	4.82 ± 0.13^ab^	0.28 ± 0.01^b^	0.18 ± 0.005^a^	0.34 ± 0.049^c^	204.87 ± 3.18^b^	55.62 ± 0.16^a^	139.98 ± 0.49^a^	99.04 ± 0.35^b^	12.2 ± 0.11^a^

*The values were presented as mean ± SE, and means represented by the same letters are not significantly different at p ≤ 0.01. Each experiment was carried out in triplicate, and each experimental unit had six plants (n = 18)*.

P-limited conditions increased the Fe content by 101.6%, as compared with the control, but the increment of Fe content was less (i.e., 74.71%, as compared with the control) in those plants grown in the ANE-supplemented P-limited media (Table 1). The application of ANE significantly increased the uptake of B by 40% in those plants grown under normal conditions. P-limited conditions increased the uptake of B in plants grown in 1/2 MS-P (T_2_) and 1/2 MS-P + 0.01% ANE (T_3_), but the addition of ANE in P-limited media significantly increased the B content by 8.0%, as compared with the plants grown in P-limited conditions. ANE treatment increased the uptake of Mn by 7.6 and 11.5% in plants grown under the normal and P-limited conditions, respectively (Table 1).

### Effects of ANE Treatment on the Pigment Content of *Z. mays* Grown Under P-Limited Conditions

**Figure 3:**
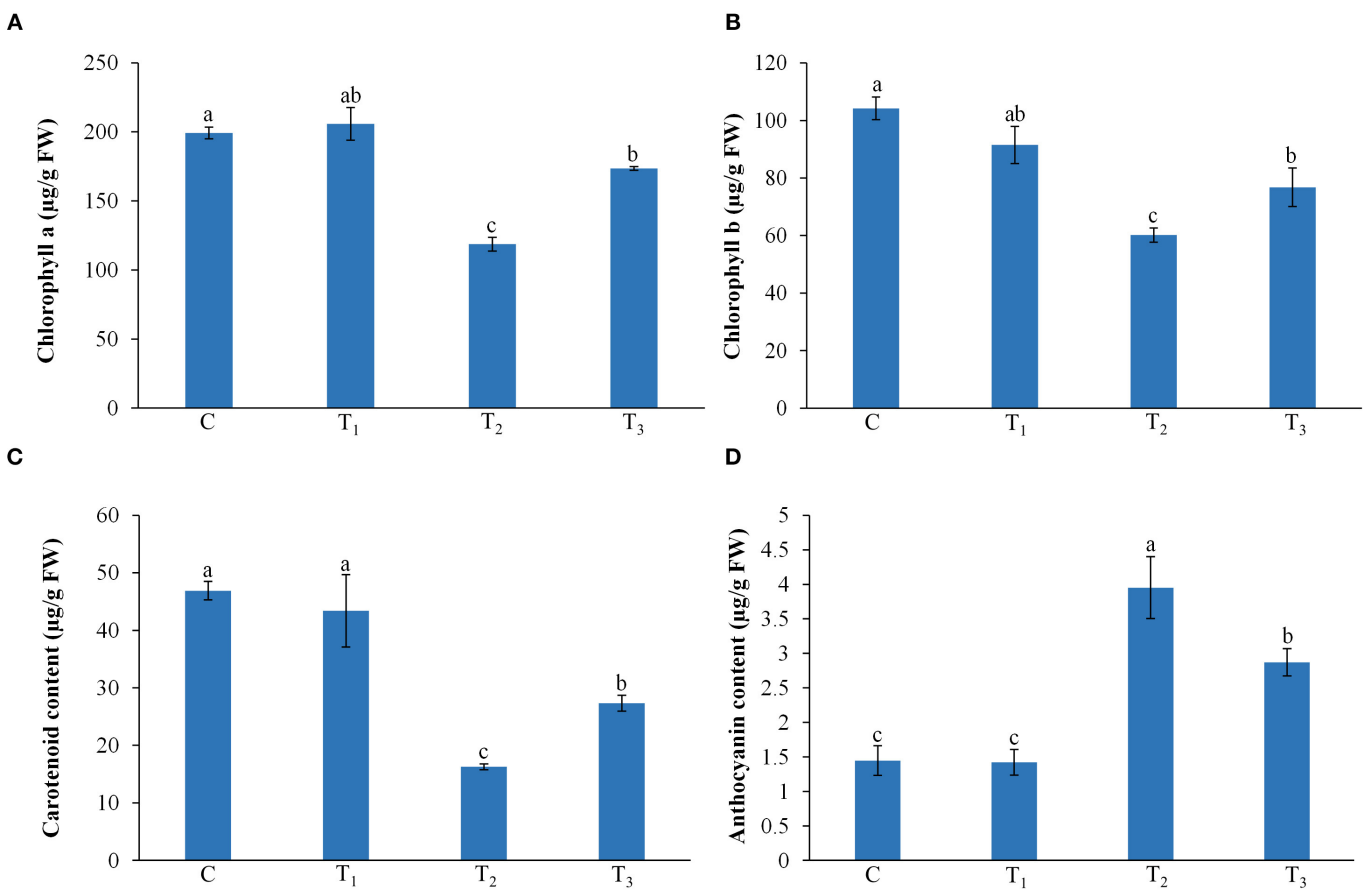
Effect of ANE on **(A)** chlorophyll a, **(B)** chlorophyll b, **(C)** carotenoid, and **(D)** anthocyanin contents of *Z. mays* grown under the P-limiting conditions. C (control): plant grown in perlite supplemented with 1/2 MS, T_1_: plant grown in perlite supplemented with 1/2 MS + ANE, T_2_: plant grown in perlite supplemented with 1/2 MS-P, T_3_: plant grown in perlite supplemented with 1/2 MS-P + ANE. The values were presented as mean ± SE, and means represented by the same letters are not significantly different at *p* ≤ 0.01. Each experiment was carried out in triplicate, and each experimental unit had six plants (*n* = 18).

ANE treatment showed no significant (*p* ≤ 0.01) effects on the chlorophyll a and b, carotenoid, and anthocyanin contents of leaves of *Z. mays* grown under normal conditions, as compared with the control (Figures 3A,B). The P-limited condition reduced the leaf content of chlorophyll a and b, whereas supplementation with ANE significantly increased the content of chlorophyll a and b in P-limited conditions. Carotenoid was also found to be higher in the ANE-treated plants grown in the P-limited media than in the plants grown in P-limited media alone (Figure 3C). The anthocyanin content was found to be increased by 173.1% as a result of the imposed P-limited conditions, whereas ANE-treated plants showed significantly less (98.3%) anthocyanin accumulation in plants grown under P-limited conditions (Figure 3D).

### Effect of ANE Treatment on Electrolyte Leakage, Membrane Stability, and MDA Contents of *Z. mays* Grown Under P-Limited Conditions

**Figure 4:**
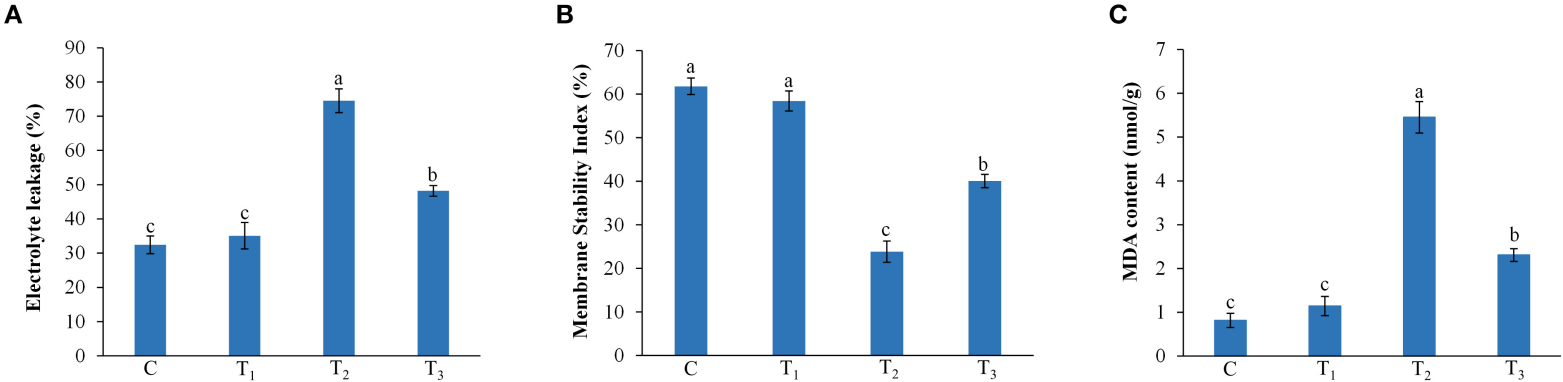
Effect of ANE on **(A)** electrolyte leakage, **(B)** membrane stability index, and **(C)** MDA contents of *Z. mays* grown under the P-limiting conditions. C (control): plant grown in perlite supplemented with 1/2 MS, T_1_: plant grown in perlite supplemented with 1/2 MS + ANE, T_2_: plant grown in perlite supplemented with 1/2 MS-P, T_3_: plant grown in perlite supplemented with 1/2 MS-P + ANE. The values were presented as mean ± SE, and means represented by the same letters are not significantly different at *p* ≤ 0.01. Each experiment was carried out in triplicate, and each experimental unit had six plants (*n* = 18).

*Z. mays* plants treated with ANE showed a significant reduction of electrolyte leakage of leaves of those plants grown under P-limitation (Figure 4A). ANE reduced the electrolyte leakage by 35.3% in the leaves of *Z. mays* grown under P-limited conditions, as compared with the plants grown under P-limited conditions alone. MSI analysis revealed that the cell membranes of plants grown under P-limited conditions were more stable in those plants supplemented with ANE (Figure 4B). Membrane stability of the plants grown in 1/2 MS-P + 0.01% ANE was 68.2% higher than that of the plants grown in 1/2 MS-P (Figure 4B). To determine the impact of ANE treatment on the membrane lipids during P-limited conditions, the MDA content was measured in leaves of *Z. mays* grown under different treatments. No significant differences were observed in the MDA content of plants grown in 1/2 MS and 1/2 MS + 0.01% ANE. Compared with those plants grown in 1/2 MS-P, the MDA content was significantly reduced by 57.6% in ANE-supplemented plants, suggesting that lipid peroxidation of membrane lipids was significantly less than those grown in P-limited conditions (Figure 4C).

### ANE Treatment Reduced Oxidative Damage Induced by P-Limited Conditions

**Figure 5:**
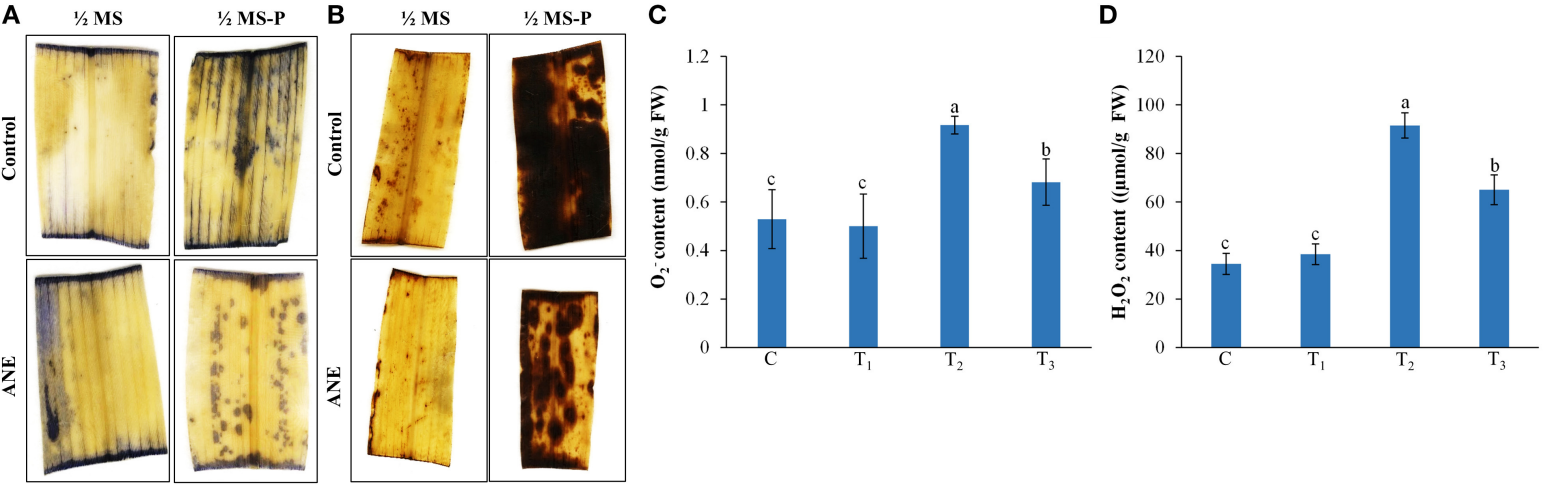
*In vivo* localization and quantification of **(A,C)** ${\text{O}}_{2}^{-}$ and **(B,D)** H_2_O_2_ in leaves of *Z. mays* grown for 21 days in perlite supplemented with 1/2 MS (C, control), 1/2 MS + ANE (T_1_), 1/2 MS-P (T_2_), 1/2 MS-P + ANE (T_3_). **(A)** Localization of ${\text{O}}_{2}^{-}$ by NBT staining, **(B)** localization of H_2_O_2_ by DAB staining, **(C)** quantification of ${\text{O}}_{2}^{-}$ content, and **(D)** quantification of H_2_O_2_ content. The values were presented as mean ± SE, and means represented by the same letters are not significantly different at *p* ≤ 0.01. Each experiment was carried out in triplicate, and each experimental unit had six plants (*n* = 18).

The impact of ANE treatments on oxidative damage induced by P-limited conditions was assessed by staining ${\text{O}}_{2}^{-}$ and H_2_O_2_ in the tissues of leaves in treated *Z. mays* plants (Figure 5). Control and ANE-treatments showed similar patterns of staining, whereas P-limited conditions showed more staining of ${\text{O}}_{2}^{-}$ and H_2_O_2_ than the ANE-treated plants (Figures 5A,B). These results suggested that ANE treatments helped plants to reduce the P-limited-induced accumulation of ${\text{O}}_{2}^{-}$ and H_2_O_2_ *in situ*. These results were further confirmed by quantification of the tissue ${\text{O}}_{2}^{-}$ and H_2_O_2_ contents (Figures 5C,D) that were significantly reduced in those ANE-treated plants grown under P-limited conditions, as compared with the control (Figures 5C,D).

### ANE Treatment Improved Total Content of Soluble Sugars, Amino Acids, Phenolics, and Flavonoids in the *Z. mays* Grown Under P-Limited Conditions

**Figure 6:**
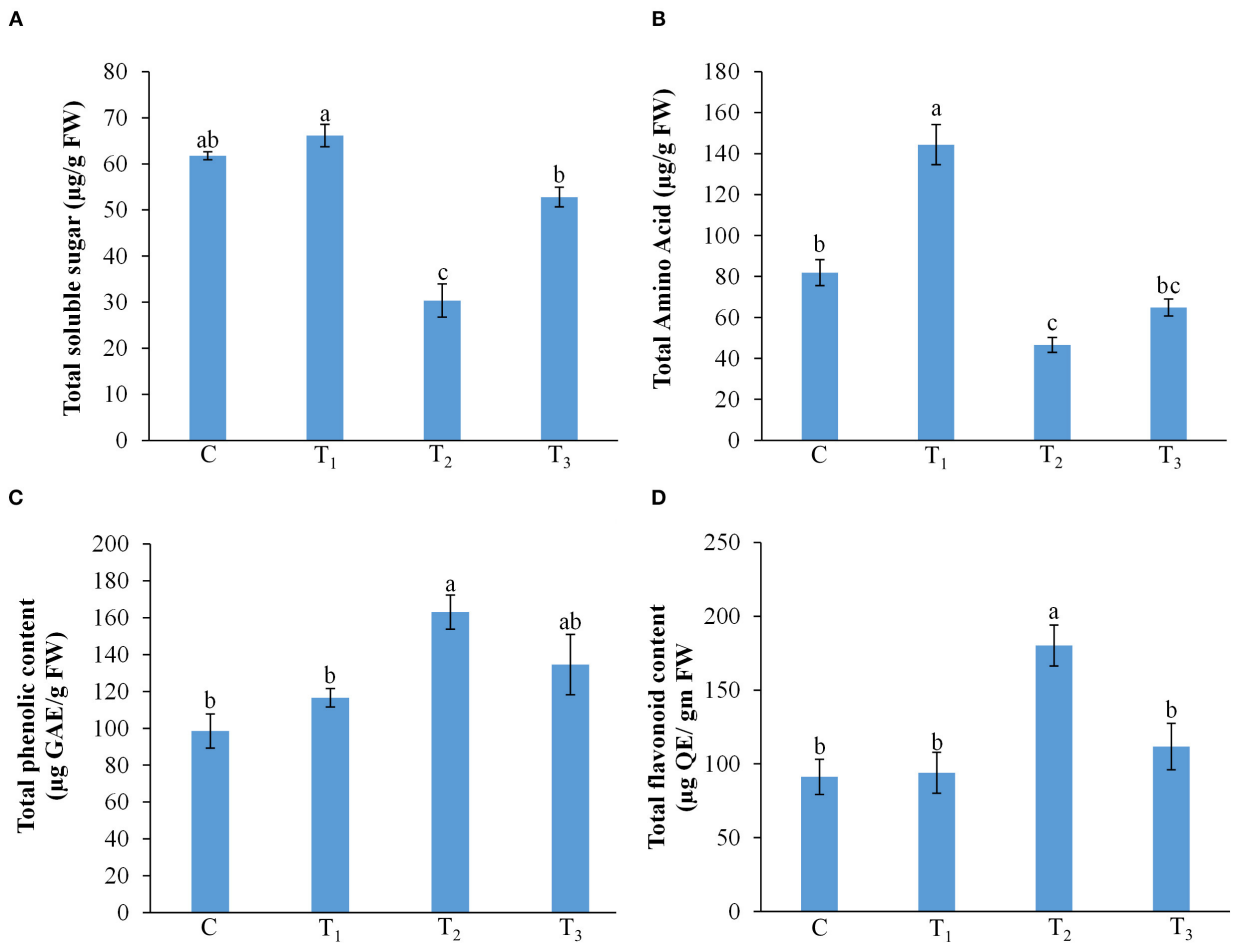
Effect of ANE on **(A)** total soluble sugar, **(B)** total amino acid, **(C)** total phenolic, and **(D)** total flavonoid content of *Z. mays* under the P-limiting conditions. C (control): plants grown in perlite supplemented with 1/2 MS, T_1_: plants grown in perlite supplemented with 1/2 MS + ANE, T_2_: plants grown in perlite supplemented with 1/2 MS-P, T_3_: plants grown in perlite supplemented with 1/2 MS-P + ANE. The values were presented as mean ± SE, and means represented by the same letters are not significantly different at *p* ≤ 0.01. Each experiment was carried out in triplicate, and each experimental unit had six plants (*n* = 18).

P-starvation is known to influence the carbohydrate metabolism in plants (Karthikeyan et al., 2007). In maize plants grown on P-sufficient media, the level of total soluble sugars remained almost the same in both the control and ANE-supplemented plants. Plants grown on a P-limited medium showed a significant reduction in total soluble sugars content (Figure 6A). The supplementation of media with 0.01% ANE in P-limited media significantly increased the total soluble sugars content by 74%, as compared with the plants grown in P-starved medium (Figure 6A). The increased levels of total amino acids content were observed in those plants grown in 1/2 MS + 0.01% ANE, as compared with the control (Figure 6B). P-limitation showed a significant (*p* ≤ 0.01) reduction in amino acid metabolites. ANE application improved total amino acids content in those plants grown in P-limited media (Figure 6B). Imposition of a P-limited condition induced the total phenolics content, whereas the addition of ANE in the P-limited media reduced the total phenolics content in the plant (Figure 6C), suggesting that ANE treatment relieved plants from the deleterious effects of P-limitation. Similarly, the total flavonoids content was also significantly reduced in plants grown in ANE-supplemented P-limited media (Figure 6D). These results suggest that ANE supplementation in P-starved media improved plant growth by modulating the biochemical status of the plants.

### Treatments With ANE Regulated the Expression of Genes Involved in P Homeostasis in *Z. mays* Grown Under P-Limited Conditions

**Figure 7:**
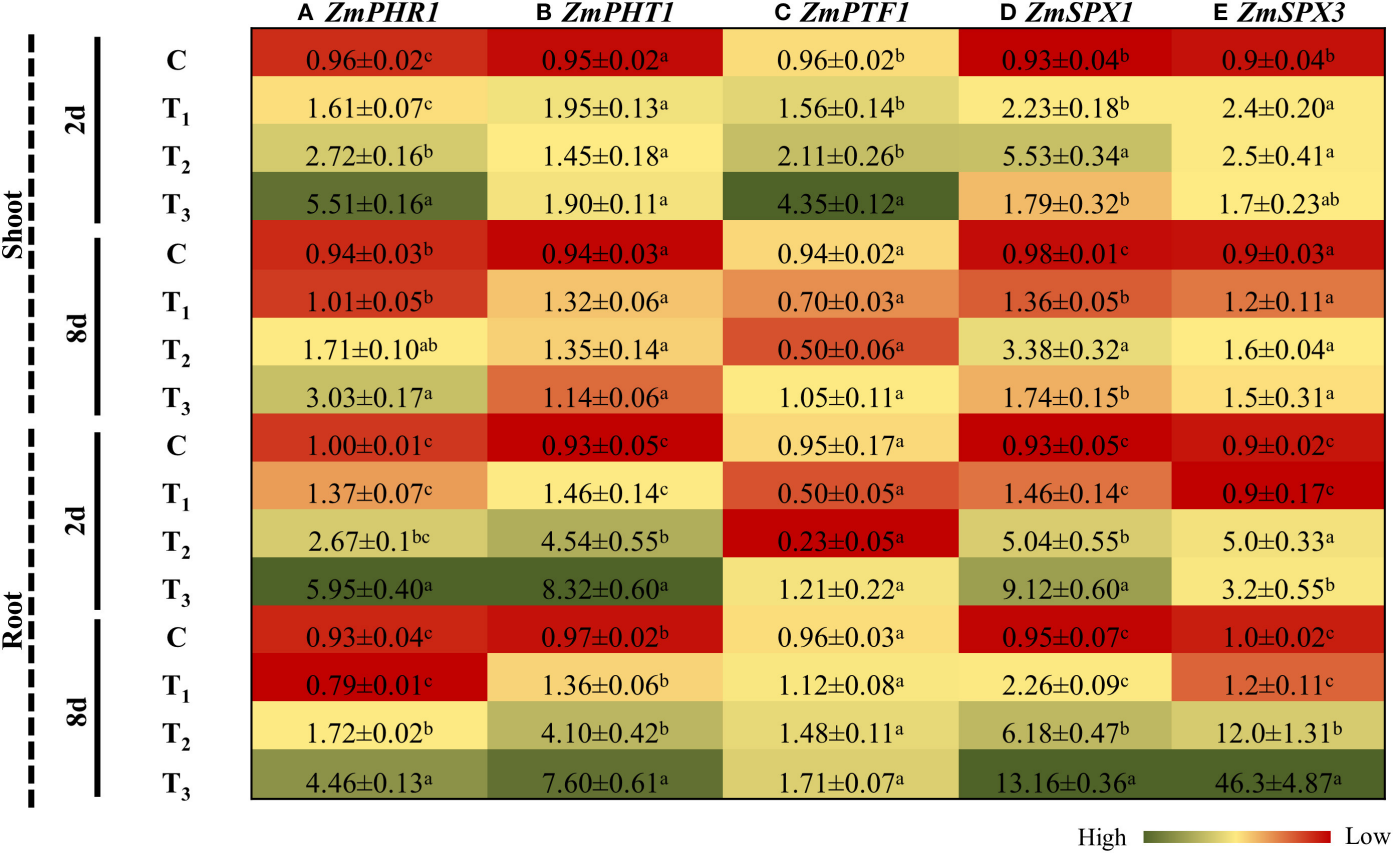
ANE altered the expression of **(A)** *ZmPHR1*, **(B)** *ZmPHT1*, **(C)** *ZmPTF1*, **(D)** *ZmSPX1*, and **(E)** *ZmSPX3* involved in phosphate homeostasis in *Z. mays* grown under P-limiting conditions. The colors ranging from red to green represent the average values of fold change (red, low expression and green, high expression). The values were presented as mean ± SE of three independent replicates, and significantly different mean values were represented by different alphabets.

To further elucidate the role of ANE treatments in ameliorating plant growth in P-impoverished conditions, the expression of genes involved in P homeostasis was evaluated in both the shoots and roots of *Z. mays* plants after 2 and 8 days of P-limited conditions. PHR1, a GARP-type MYB transcription factor, was significantly induced in the shoots and roots of the control, as well as ANE-supplemented plants grown under P-limited conditions, at both time-points investigated (Figure 7A). The induction of PHR1 in the roots and shoots of ANE-supplemented plants was significantly less (*p* ≤ 0.01) than that of the control. No significant change in the expression of *PHT1* was observed in the shoots of *Z. mays* grown in the different treatments (Figure 7B). After 2 and 8 days, *PHT1* was significantly induced in the roots of plants grown in 1/2 MS-P and 1/2 MS-P + ANE. The addition of ANE in P-limited media limited the transient up-regulation of PHT1, as observed in those plants grown under P-limitation alone (Figure 7B). PTF1, a P-starvation-induced, basic helix–loop–helix (bHLH) transcription factor, was induced in shoots after 2 days of imposition of P-limited conditions in treatments supplemented with ANE vs. control (Figure 7C). After 8 days, ANE treatments showed a reduction in the expression of *PTF1* (Figure 7C). In roots, P-limited conditions induced the expression of PTF1 at both time-points, whereas ANE supplementation in P-starved media showed reductions in the expression of *PTF1* (Figure 7C). These results suggested that experimental supplementation with ANE reduced the physiological severity of P-limitation.

SPX (SYG1/Pho81/XPR1) genes are known to play a key role in maintaining P homeostasis in plants (Secco et al., 2012). The expression of *SPX1* was significantly induced in ANE-supplemented plants, as compared with the controls. From day 2 to day 8 of P-limitation, the expression of *SPX1* was reduced from 5.94 times to 3.44 times in the shoots of *Z. mays* (Figure 7D). However, no change was observed in the expression of *SPX1* in ANE-supplemented plants across the time-points. After 2 and 8 days of application of ANE, the expression *SPX1* was increased by 1.46 and 2.26 times in the roots (Figure 7D). Under P-limited conditions, the *SPX1* transcript was strongly expressed (5.04 times) at day 2, which was further increased to 6.18 times after 8 days of treatment. However, the addition of ANE in the 1/2 MS-P highly induced the expression of *SPX1* in the roots at both time-points (Figure 7D). The expression of *SPX3* remained unaltered in the shoots of plants grown in different treatments at both time-points. Similarly, plants grown in 1/2 MS (C) and 1/2 MS + ANE (T_1_) did not show any change in the expression of *SPX3* in their roots, across different time-points. P-limitation consistently increased the expression of *SPX3* in roots, at both time-points. Overall, under P-limitation, the application of ANE resulted in strong induction (14.5 times) across the time-points, whereas P-limited conditions alone showed only 2.4 times induction from days 2 to 8 after treatment (Figure 7E).

### ANE Treatment Regulated the Expression of Genes Involved in Carbohydrate Metabolism in the *Z. mays* Grown Under P-Limited Conditions

**Figure 8:**
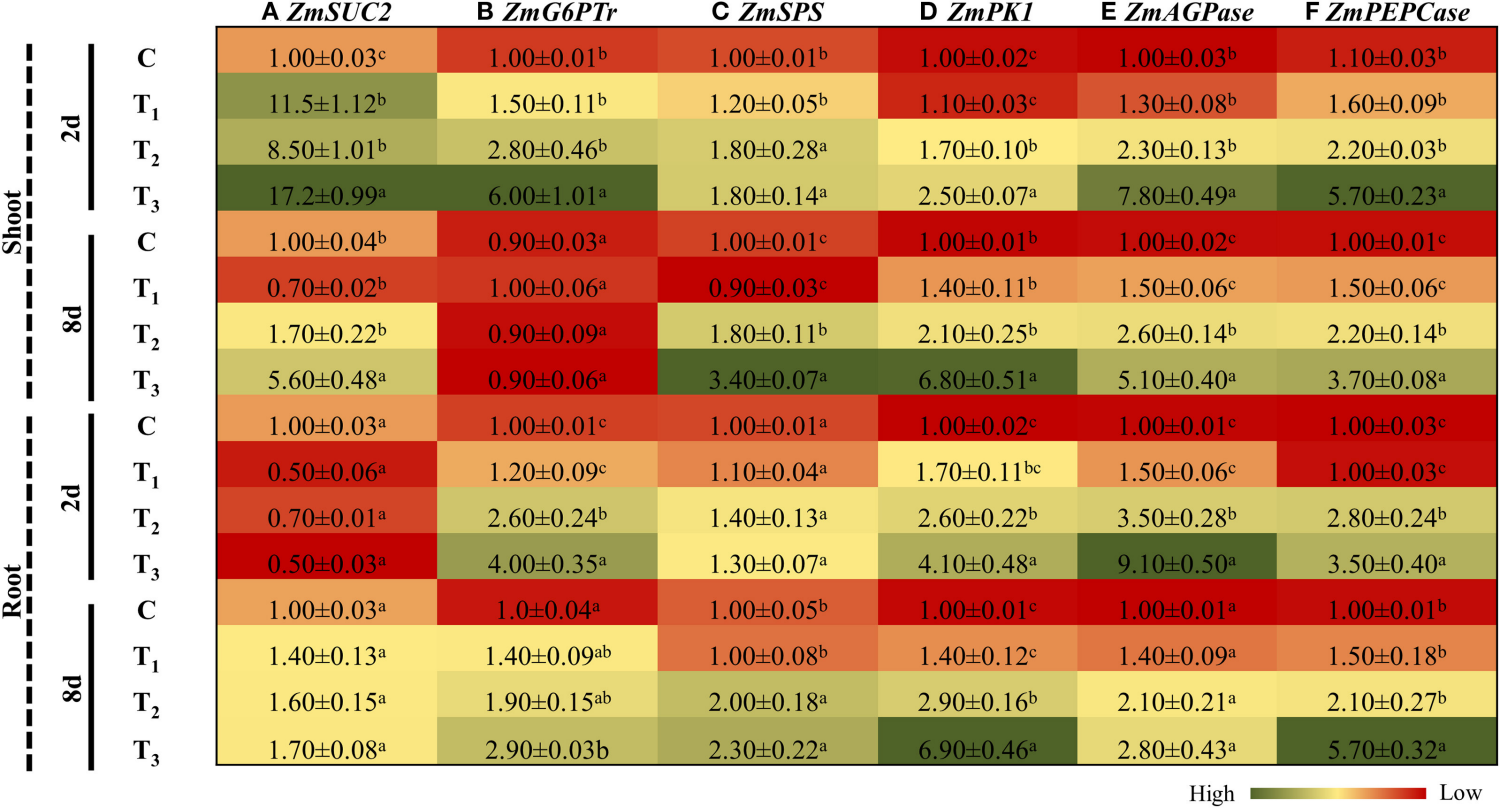
ANE regulated the expression of **(A)** *ZmSUC2*, **(B)** *ZmG6PTr*, **(C)** *ZmSPS*, **(D)** *ZmPK1*, **(E)** *ZmAGPase*, and **(F)** *ZmPEPCase* involved in carbohydrate metabolism in *Z. mays* grown under P-limiting conditions. The colors ranging from red to green represent the average values of fold change (red, low expression and green, high expression). The values were presented as mean ± SE of three independent replicates, and significantly different mean values were represented by different alphabets.

P-starvation had a direct impact on photosynthesis and consequently reduced the carbon assimilation in those treatments. To cope with this situation, plants maneuver the expression of different genes involved in carbohydrate metabolism (Hermans et al., 2006). In shoots, under control conditions, ANE applications resulted in a significant induction (11.5 times) in the expression of *SUC2*, a gene involved in organ-specific sucrose transportation. After 2 days of imposition of P-limited conditions, the expression of *SUC2* induced the expression by 8.5 times in the shoots, as compared with the control, whereas supplementation of ANE in the P-limited media further induced the expression by 17.2 times. However, after 8 days, the expression pattern of *SUC2* was found to be 3.18 times higher in the shoots of ANE-supplemented plants grown in P-limited media than in those plants grown in P-limited media alone (Figure 8A). In the roots, initially, after 2 days, *SUC2* was down-regulated in all treatments, as compared with the control, whereas at the later stage, all plants showed induction of *SUC2* in all treatments, as compared with the control (Figure 8A). The Glucose 6P/P translocator (G6Ptr) involved in the conversion of Glucose 6P to starch was differentially regulated in the shoots and roots of *Z. mays* grown under P-limited conditions (Figure 8B). After 2 days of P-limitation, ANE supplementation induced the expression of G6Ptr in the shoots (Figure 8B), whereas after 8 days, no differences in expression patterns were observed in any of the treatments. In roots, the transcript accumulation of *G6Ptr* was found to be higher at both time-points in those *Z. mays* plants that were treated with ANE and grown under P-limited conditions than the control (Figure 8B). Initially, no changes in the expression patterns of sucrose phosphate synthase (*SPS*) were observed in the shoots and roots of ANE-treated, P-limited plants. However, after 8 days of P-limitation, ANE-supplemented plants showed a significant induction in the expression of *SPS*, as compared with the control (Figure 8C). ANE applications significantly stimulated the expression of pyruvate kinase 1 (*PK1*) in the roots and shoots of plants grown under P-limited conditions, at both time-points, as compared with the plants grown in P-limited media alone (Figure 8D). ADP-glucose pyrophosphorylase (*AGPase*) was significantly induced in the shoots of ANE-treated, P-limited plants at both time-points. In the roots, after 2 days of P-limitation, the expression of *AGPase* was 2.6 times higher in the ANE treatments, which declined to 1.33 times after 8 days, than in the control (Figure 8E). Likewise, phosphoenolpyruvate carboxylase (PEPCase), an important gene involved in carbon assimilation, was found to be significantly up-regulated in the shoots and roots of ANE-supplemented, P-limited plants, at both time-points (Figure 8F). These results suggest that the application of ANE regulated the carbon assimilation and better plant growth resulted under P-limited conditions.

### ANE Treatment Regulated the Expression of Genes Involved in Lipid Metabolism in *Z. mays* Grown Under P-Limited Conditions

**Figure 9:**
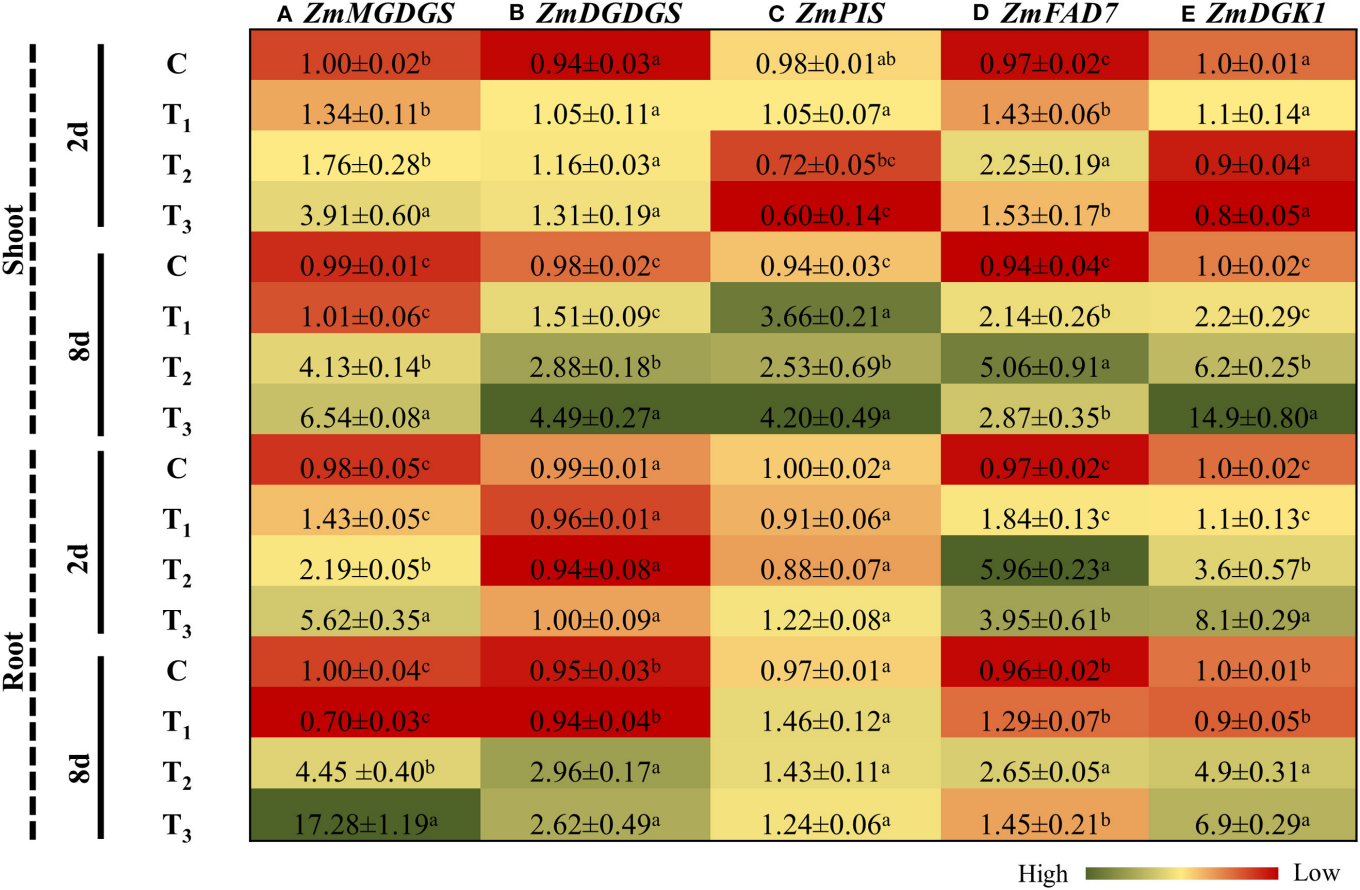
ANE altered the expression of **(A)** *ZmMGDGS*, **(B)** *ZmDGDGS*, **(C)** *ZmPIS*, **(D)** *ZmFAD7*, and **(E)** *ZmDGK1* involved in lipid metabolism in *Z. mays* grown under P-limiting conditions. The colors ranging from red to green represent the average values of fold change (red, low expression and green, high expression). The values were presented as mean ± SE of three independent replicates, and significantly different mean values were represented by different alphabets.

To survive in P limiting conditions, plants go through significant alterations in their membrane lipid compositions by regulating the expression of different genes involved in lipid metabolism. Monogalactosyldiacylglycerol (MGDG) and digalactosyldiacylglycerol (DGDG) are the main components of glycolipids of the thylakoid membrane. MGDG synthase (MGDGS) and DGDG synthase (DGDGS) catalyze the formation of MGDG and DGDG. P-limited conditions induced the expression of *MGDGS* and *DGDGS* in the shoots and roots of *Z. mays* at both time-points (Figures 9A,B). ANE supplementation increased the expression of *MGDGS* in the shoots of *Z. mays* grown under P-limited conditions, by 2.6 and 1.58 times at 2 and 8 days, respectively. Similarly, in the roots, the expression of *MGDGS* was significantly induced at both time-points in ANE-treated, P-limited plants (Figure 9A). After 2 days of P-limited conditions, no changes in the expression pattern of *DGDGS* were observed in either the shoots or roots of the treatments (Figure 9B). Prolonged exposure of P-limited conditions in the presence of ANE increased the transcript accumulation of *DGDGS* in shoots, whereas no substantial differences were observed in the roots, as compared with the control (Figure 9B). Phosphatidylinositol (PIS) is a key enzyme involved in phospholipid pathways. After 2 days, the expression of PIS was significantly reduced in plants grown in 1/2 MS-P (T_2_) and 1/2 MS-P + ANE (T_3_), as compared with those plants grown in 1/2 MS (C) and 1/2 MS + ANE (T_1_) (Figure 9C). ANE significantly induced the expression of *PIS* after 8 days, as compared with the control plants (Figure 9C). P-limited conditions, however, increased the expression of *PIS* from 0.7 to 2.5 times from day 2 to 8, whereas the treatment of ANE application in P-starved media increased expression from 0.6 to 4.2 times from day 2 to 8 (Figure 9C). Notably, in the roots, no difference was observed in the expression of *PIS* among all the treatments at both time-points (Figure 9C). In the shoots of those plants grown under P-limited conditions, the expression of fatty acid desaturase 7 (*FAD7*) increased from 2.2 to 5.1 times between time-points. This trend was not observed in plants grown under P-limited conditions with ANE treatment (Figure 9D). In roots, *FAD7* was also induced at both the time-points by P-limited conditions. By contrast, the expression was reduced from 6 to 2.6 times from day 2 to 8 (Figure 9D). In the roots of ANE-treated plants, under P-limited condition, *FAD7* was also induced at both time-points, but to a lesser degree than those plants grown in P-limited conditions (Figure 9D). The expression of diacylglycerol kinase 1 (*DGK1*) was significantly induced (2.2 times) in the shoots of plants treated with ANE, as compared with the control conditions (Figure 9E). P-limited conditions increased the expression of *DGK1* in the shoots of plants grown with ANE, as well as without ANE, but the expression was 2.81 times higher in ANE-supplemented plants grown under P-limited conditions than in the plants grown under P-limited conditions alone (Figure 9E). In roots, *DGK1* was significantly induced at both time-points by P-limited conditions; however, ANE supplementation in P-starved media induced *DGK1* by 2.25 times and 1.4 times at 2 and 8 days, respectively (Figure 9E). These results suggest that ANE supplementation regulated the expression of genes involved in lipid metabolism that helped treated plants to grow better under P-limited conditions.

### ANE Treatment Regulated the Expression of Genes Involved in Secondary Metabolism in *Z. mays* Under P-Limited Conditions

**Figure 10:**
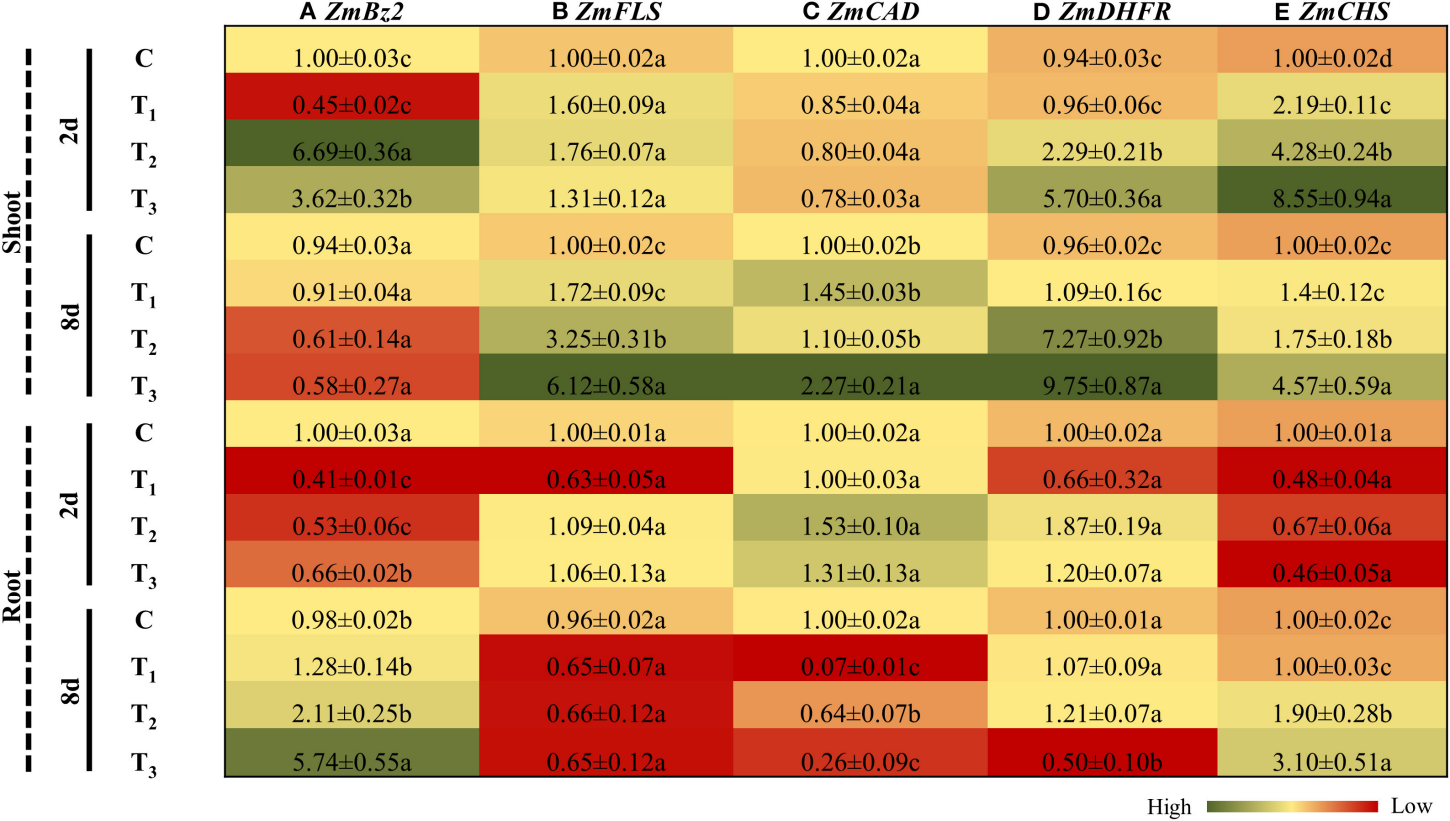
ANE altered the expression of **(A)** *ZmBz2*, **(B)** *ZmFLS*, **(C)** *ZmCAD*, **(D)** *ZmDHFR*, and **(E)** *ZmCHS* involved in secondary metabolism in *Z. mays* grown under P-limiting conditions. The colors ranging from red to green represent the average values of fold change (red, low expression and green, high expression). The values were presented as mean ± SE of three independent replicates, and significantly different mean values were represented by different alphabets.

After 2 days of treatment, *Bronze 2* (*Bz2*), an anthocyanin biosynthetic gene in maize, was significantly down-regulated (2 times) in the shoots of plants grown in the 1/2 MS media supplemented with ANE, as compared with the control plants (Figure 10A). *Bz2* was induced by 1.86 times in plants grown for 2 days in 1/2 MS-P (T_2_), as compared with the plants grown in 1/2 MS-P + ANE (T_3_) (Figure 10A). The expression of *Bz2* was reduced in the shoots after 8 days under P-limited conditions. However, no changes were observed in shoots of the ANE-treated plants under P-limited conditions (Figure 10A). In roots, after 2 days of treatment, *Bz2* was down-regulated in all treatments, as compared with the control, whereas maximum reduction in expression was observed in those plants grown in 1/2 MS + ANE (T_1_) (Figure 10A). Prolonged exposure of P-limited conditions increased the expression of *Bz2* in those plant roots. Addition of ANE in P-limited media significantly increased the expression of *Bz2* in the roots by 2.7 times, as compared with the plants grown under P-limited conditions alone (Figure 10A). Flavonol synthase 1 (*FLS1*), an important gene involved in flavonol biosynthesis, was differentially regulated in the shoots and roots of *Z. mays* in all treatments (Figure 10B). Initially, no change in the expression of *FLS1* was observed (Figure 10B). However, after 8 days under P-limited conditions, ANE-supplemented plants showed 1.8 times increased expression of *FLS1*, as compared with the P-limited plants alone (Figure 10B). No significant changes were observed in the expression of *FLS1* in the roots of plants grown under different treatments, at both time-points (Figure 10B). The expression of cinnamyl alcohol dehydrogenase (*ZmCAD*) remained unchanged in the shoots and roots of *Z. mays* in all treatments after 2 days (Figure 10C). Expression analysis revealed that the transcript accumulation of CAD increased in the shoots of ANE-supplemented plants grown for 8 days under P-limited conditions. In roots at 8 days after treatment, expression was significantly reduced in the ANE-treated plants grown under P-limited conditions (Figure 10C). Under P-limited conditions, ANE supplementation significantly increased the expression of dihydroflavonol-4-reductase (*ZmDHFR*) and chalcone synthase (*ZmCHS*) in the shoots of *Z. mays* at both time-points (Figures 10D,E). These results allow us to draw a conclusion that ANE regulates secondary metabolism of plants grown under P-limited conditions and help the plant to reduce the deleterious effect of P-limitation.

## Discussion

In order to provide basic food security for an ever-increasing global population, chemical fertilizers have been used excessively to increase agricultural productivity (Savci, 2012a). Excessive use of chemical fertilizers acts as an agricultural pollutant (Savci, 2012a,b) and is a significant anthropogenic threat to the world's terrestrial and aquatic ecosystems. Nevertheless, P is an important soil nutrient required for the maintenance of plant growth and productivity and is generally supplied in the form of chemical fertilizer (Kim et al., 2018). To address this challenge, there is a compelling need to develop sustainable strategies that enhance the agricultural output, while minimizing the chemical input.

**Figure 11:**
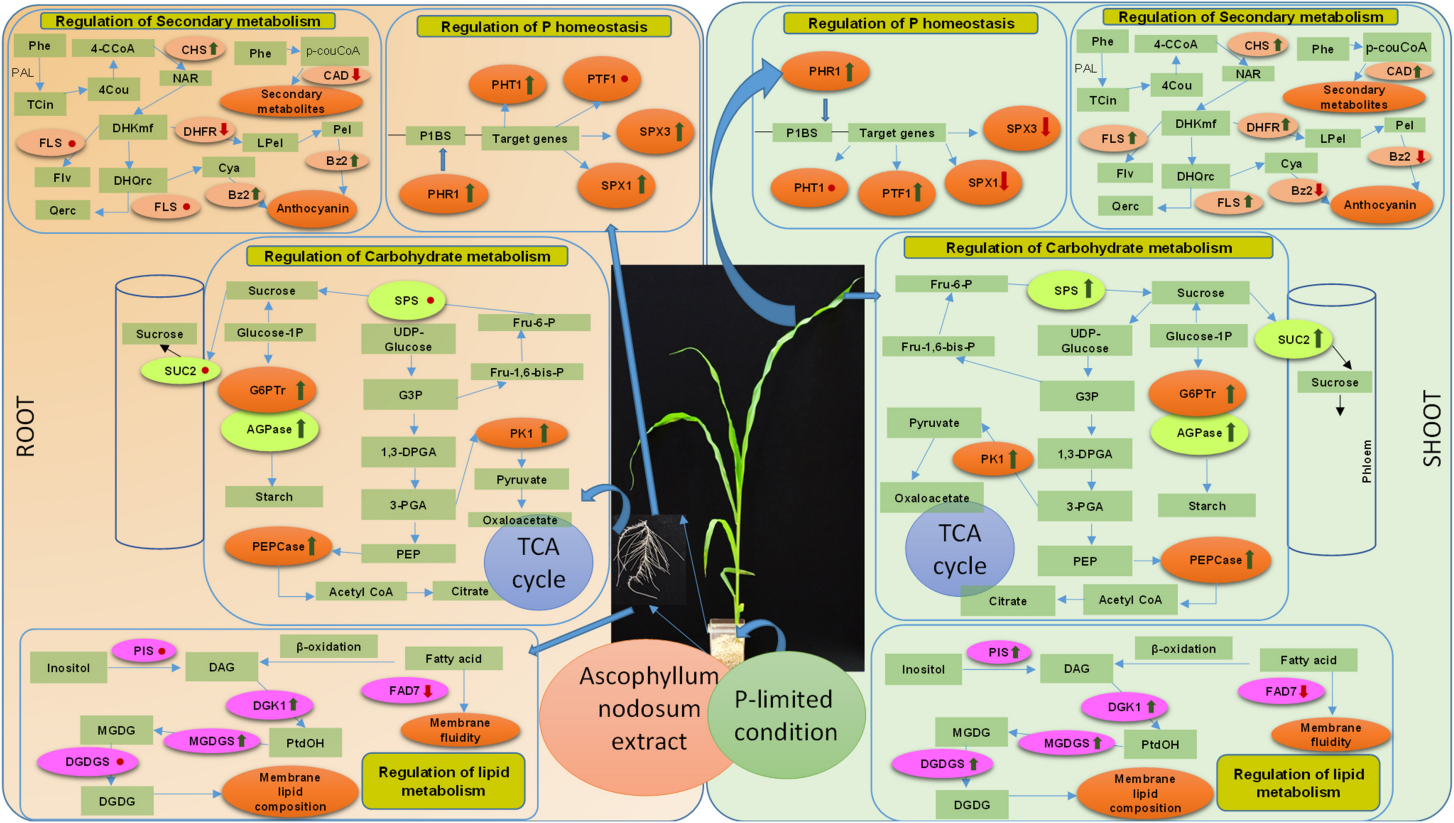
A schematic presentation of regulation of the genes involved in P homeostasis, lipid, carbohydrate, and secondary metabolism by the *A. nodosum* extract in the shoots and roots of the *Z. mays* grown under P-impoverished conditions. The green arrow shows the up-regulation of the gene expression, whereas the red arrow shows the down-regulation. The red dot represents no significant change in the expression. Abbreviations listed in the diagram are as follows: P homeostasis (PHR1, Phosphate Starvation Response 1; PTF1, basic helix–loop–helix domain containing transcription factor; SPX1 and SPX3, SYG1/Pho81/XPR1 domain containing protein; PHT1, Pi transporters), carbohydrate metabolism (SUC2, sucrose transporter; SPS, sucrose phosphate synthase; PEP, phosphoenolpyruvate; AGPase, ADP-glucose pyrophosphorylase; G6Ptr, Glucose 6P/P translocator; PK, pyruvate kinase 1; PEPCase, phosphoenolpyruvate carboxylase; G3P, Glyceraldehyde-3-phosphate; 1,3-DPGA, 1,3 di-phosphoglyceraldehyde; 3-PGA, 3-phosphoglyceraldehyde; Fru 6-P, fructose-6-phosphate; Fru-1,6-bis-P, fructose 1,6-bis-phosphate), secondary metabolism (Phe, phenylalanine; TCin, transcinnamate; 4Cou, 4-coumarate; 4-CCoA, 4-coumaroyl CoA; Nar, naringenin; DHKmf, dihydrokaempferol; Flv, flavonoids. DHQrc, dihydroquercetin; Qerc, quercetin. Lpel, leucopelargonidin. Cya, cyanidin; Pel, pelargonidin; PAL, phenylalanine ammonia lyase; CHS, chalcone synthase; FLS, flavonol synthase; BZ2, glutathione-S-transferase encoded by Bronze 2; DFR, dihydroflavonol-4-reductase; pcouCoA, p-coumaroyl-CoA; CAD, cinnamyl alcohol dehydrogenase).

In this study, we report that judicious applications of an alkaline extract of ANE are sustainable strategies to improve the growth of *Z. mays* grown under P-impoverished conditions. The ICP-OES analysis reveals the presence of 5.0 μM of P in 0.01% ANE (Supplementary Table 2), and the same amount of P was added to 1/2 MS-P to construct a positive control that would define the role of ANE in ameliorating the growth of plant in P-limited conditions. ANE improved leaf area, root length, and fresh and DW of shoots and roots of corn grown under P-limited conditions. The application of ANE showed increased uptake of Mg, B, Mn, Zn, and Cu in those shoots grown under P-limited conditions. Similarly, Di Stasio et al. (2018) also reported that two commercial extracts, Rygex® and Super Fifty®, also prepared from *A. nodosum*, enhanced the nutrient contents of tomato fruits. Additionally, the supplementation of ANE in P-limited media reduces Fe content in plants, as compared with the plants grown in P-limited media alone. Briat et al. (2015) explained the physiological connections between reductions in Fe content in plants grown under P-limitation. ANE-supplemented plants grown under P-limited conditions showed higher P content than plants grown in P-limited media alone, which demonstrated the “biostimulant boost” of ANE, as opposed to it acting as a micro- or macronutrient fertilizer alone. To further explain this behavior, we conducted expression analysis of genes involved in P homeostasis. ANE differentially up-regulated the expression of *ZmPHR1* in those shoots and roots of *Z. mays* grown under P-limited conditions. In the present study, ANE up-regulated the expression of *PHR1* by down-regulating the expression of *SPX1* in the shoots grown under P-limited conditions (Figure 11). PHR1, a MYB transcription factor, is known to control a large part of P-starvation responsive transcriptome (Nilsson et al., 2007; Pant et al., 2015), and its expression is negatively regulated by SPX1 and SPX3 (Puga et al., 2017). In addition to its role in P homeostasis, PHR1 also controls the integration of P and Fe homeostasis (Briat et al., 2015). High P availability down-regulates the expression of PHO2 which in turn induces the expression of PHT1, phosphate transporter genes expressed in the roots of plant (Doerner, 2008). ANE supplementation also induced the expression of PHT1 in the roots of plants grown under P-limitation, whereas its expression remained unchanged in the shoots. ANE treatment also modulated the expression of *PTF1*, a bHLH transcription factor involved in tolerance to P-limited conditions (Li et al., 2011). Similarly, Shukla et al. (2018) showed that the application of ANE in P-starved media improves the growth of *Arabidopsis*. ANE is also reported to increase P uptake in *Arabidopsis* grown under saline conditions by regulating the expression of regulatory RNAs and genes involved in the efficient relocation of P resources (Shukla et al., 2018, 2019). Taken together, these results suggest that applications of ANE improved the growth of *Z. mays* by regulating the expression of genes involved in P homeostasis (Figure 11).

To sustain growth in P-limited conditions, plants have developed a number of developmental and metabolic responses to adapt to both the internal P status in plants and the external soil P availability (Jiang et al., 2007; Pant et al., 2014). P-limited conditions reduce the chlorophyll content in the plants (Manna et al., 2015). The supplementation of ANE in P-starved media increased chlorophyll a and b and carotenoid contents of *Z. mays*. Contrary to the chlorophyll content, P-limited conditions contributed to anthocyanin accumulation (Jiang et al., 2007), whereas the accumulation was less in ANE-supplemented plants grown under P-limited conditions. Phosphorous limitation has a direct impact on photosynthesis and leads to reduced carbon assimilation (Rosa et al., 2009; Lemoine et al., 2013). It is well-known that P deficiency reduces the sugar and starch accumulation in the leaves of plants by regulating the expression of those genes involved in photosynthesis and sucrose biosynthesis (Cakmak et al., 1994; Hermans et al., 2006). In the present study, the application of ANE in P-limited media increased the total soluble sugars by regulating the expression of genes involved in carbohydrate metabolism (Figure 11). Under P-limited conditions, plants reallocate their carbon sources to their roots by increasing the loading of sucrose to the phloem through regulating the expression of SUC2, a sucrose transporter involved in phloem loading (Lloyd and Zakhleniuk, 2004; Hammond and White, 2008). ANE supplementation increased sucrose translocation to the roots by increasing the expression of *SUC2* in the shoots of plants grown under P-limited conditions. However, expression was unaltered in the roots. In addition, the expression of SPS was also found to be highest in ANE-supplemented plants, grown under P-limitation. Low P activated the expression of SPS (Morcuende et al., 2007; Rosa et al., 2009), which was further enhanced by the addition of ANE in the P-limited media. ANE supplementation modulated the expression of G6PTr in the shoots, in response to P-limitation. G6PTr and AGPase are involved in the conversion of glucose-1P to starch and are reported to be differentially regulated in response to P-limited conditions (Hermans et al., 2006; Calderon-Vazquez et al., 2008). P-limited conditions regulate the allosteric regulation of AGPase in starch synthesis (Hermans et al., 2006). ANE increased the expression of AGPase in both the shoots and roots, in response to P-limitation, at both time-points. Metabolites involved in carbon assimilation were also reduced in P-starved plants (Morcuende et al., 2007). The expression of pyruvate carboxylase (*PEPCase*) and *PK1* was induced by ANE in response to P-limited conditions. Thus, ANE supplementation showed an increase in the glycolytic intermediates in those plants grown under P-limitation which, in turn, increased CO_2_ assimilation. These results provide clear evidence that ANE treatment not only helped to relocate carbohydrate resources within plants but also has the potential to regulate sugar signaling cascades that assist treated plants to improve growth under P-impoverished conditions.

Low phosphorous availability induces photo-oxidative damage in plants (Hernández and Munné-Bosch, 2015). P-limited conditions in plants lead to alterations of the photosynthetic apparatus, including reduction in carbon fixation, leading to the generation of reactive oxygen species (Hernández and Munné-Bosch, 2015). In this series of experiments, those plants treated with ANE reduced the accumulation of P-limited-induced reactive oxygen species. In addition to this, ANE-supplemented plants also showed the reduction in P-limited-induced electrolyte leakage by improving membrane stability. Several reports showed that low P availability increased the MDA content, a biomarker of lipid peroxidation in plants grown under P-limited conditions (Zhang et al., 2014). ANE supplementation reduced the accumulation of MDA content in plants grown under P-limited conditions. Membrane lipid remodeling is one of the most important metabolic adaptations in response to P-starvation (Nakamura et al., 2009; Pant et al., 2015). In an adaptive response to P-starvation, plants promote the re-mobilization of internal P by replacing the composition of membrane lipids rich in phospholipids with non-phosphorous glycerolipids, such as MGDG and DGDG (Nakamura et al., 2009; Nakamura, 2013). ANE supplementation triggered the lipid remodeling response during P-limitation by inducing the expression of *MGDGS* and *DGDGS*, key genes involved in their synthesis from diacylglycerol (DAG). Similarly, Calderon-Vazquez et al. (2008) also demonstrated that P-starvation differentially modulated the expression of the genes involved in lipid metabolism. Phosphatidylinositol synthase (*PIS*) and *DGK1*, important genes involved in phospholipid metabolism who act as phospholipid signaling molecules (Liu et al., 2013), were also differentially regulated by ANE in those plants grown under P-starvation. Plant fatty acid desaturases catalyze the desaturation of fatty acids and play an important role in regulating fatty acid composition that maintains membrane fluidity (Zhao et al., 2019). ANE altered the saturation pattern of membrane lipids by regulating the expression of FAD7. Furthermore, ANE treatments reduced the expression of FAD7 in plants grown under P-limited conditions. These results suggest that to conserve phosphorus in situations of P-limitation, ANE remodeled membrane phospholipids and replaced them by increased biosynthesis of galactolipids (Ticconi and Abel, 2004).

Accumulation of secondary metabolites are typical P-starvation responses that recycle significant amounts of P from phosphorylated precursors (Ticconi and Abel, 2004; Malusà et al., 2006). ANE supplementation increased the total amino acid content in the leaves of those corn plants grown under P-limited conditions. Low P availability is reported to differentially reduce the amino acid content and increase flavonoid content in *Camellia sinensis*. P-starvation induces the accumulation of flavonoids and phenolics in plants (Stewart et al., 2001; Trejo-Téllez et al., 2019). ANE treatment showed a reduction in the P-limitation-induced accumulation of anthocyanin, flavonoids, and phenolics contents in the corn. *Bronze*-2 (*Bz2*) plays an important role in the anthocyanin biosynthetic pathway and is significantly induced under P-limited conditions in the roots of maize (Nash and Walbot, 1992; Calderon-Vazquez et al., 2008; Calderón-Vázquez et al., 2011). The expression of *Bz2* was reduced at the early time-point in the shoot of ANE-treated *Z. mays* grown under P-limited conditions, whereas its expression was induced by ANE in the roots after the prolonged exposure of P-limitation. Similarly, ANE differentially regulated the expression of flavonol synthase (FLS), cinnamyl alcohol dehydrogenase (CAD), dihydroflavonol-4-reductase (DHFR), and chalcone synthase (CHS) in the shoots and roots of *Z. mays* grown under P-limited treatments (Figure 11).

## Conclusion

The largely undetermined bioactive components present in the chemically complex, crude commercial extract of ANE improved the growth of *Z. mays* plants grown under P-limited conditions by efficiently relocating the internal P reserves. This was achieved by regulating the physiological and biochemical processes in response to P-limited conditions. In conclusion, this study provided a holistic understanding regarding the mode of action of ANE in improving plant growth under P-limited conditions. This study provides the basis for the applications of ANE as a component of an important sustainable strategy to reduce the input of chemical fertilizers, especially those impoverished in P to agricultural farmlands. The results documented here are encouraging but to realize the full potential of ANE in nutrient management programs to reduce dependency on chemical fertilizers, more comprehensive field trials under natural conditions are required.

## Data Availability Statement

The raw data supporting the conclusions of this article will be made available by the authors, without undue reservation.

## Author Contributions

PS and BP conceived the idea, design of the experiments, drafted all versions of the manuscript, and contributed to the final versions of the manuscript. PS performed the experiments, performed data processing, analysis, and interpretation.

## Conflict of Interest

The authors declare that the research was conducted in the absence of any commercial or financial relationships that could be construed as a potential conflict of interest.
