# Supplementary material for: Ascophyllum nodosum Biostimulant Improves the Growth of Zea mays Grown Under Phosphorus Impoverished Conditions
Source: Front Plant Sci. 2021 Jan 8;11:601843. doi: 10.3389/fpls.2020.601843 (PMC7820112; doi:10.3389/fpls.2020.601843)
Supplement: Supplementary file 1 [file Data_Sheet_1.pdf]

Supplementary Material

Ascophyllum nodosum biostimulant improves the growth of *Zea mays* grown under phosphorus impoverished conditions

Pushp Sheel Shukla[1], Balakrishnan Prithiviraj[1*]

*** Correspondence:**

Balakrishnan Prithiviraj

Email: bprithiviraj@dal.ca

Telephone No: +1(902) 893-6643

Supplementary Table 1: List of the primers used in target gene expression analysis.

	Genes	Primer sequence
1.	*ZmPHT1*	F-5'- TACTGGGACTTCGTCAAGGA -3'; R-5'- GTTCAGTCCCATTGACAGGTAG-3'
2.	*ZmPHR1*	F-5'- CCAACTCAGCTACATCACTACC -3'; R-5'- GGTCCATCTCATCCGTTGTT-3'
3.	*ZmPTF1*	F-5'- TCAAGGTGTTGAGCATGAGTAG -3'; R-5'- TTGGTTGGAGCTGACAGAAG-3'
4.	*ZmSPX1*	F-5'-GTCTCGGTCGTCGGAATTG-3'; R-5'- GTTCTCCAGCAAGACCATCTC-3'
5.	*ZmSPX3*	F-5'-GGGAGGAGGACTTCGTCATA-3'; R-5'-AGTTGATGGCGCTATAGTTGAG-3'
6.	*Zm SUC2*	F-5'-AGACCCATCTACCCTAGAGAAA-3'; R-5'-CTGTGTCTGGCAAGCCTAATA -3'
7.	*Zm SPS*	F-5'-GGCAGCACCATGAACAAATAC-3'; R-5'-GATCACCTGCAGCATCTTCT-3'
8.	*Zm G6P*	F-5'-CCAGTCTCAAACCCTCCAAATA-3'; R-5'-AGCAGAACAACGGAACTCAA-3'
9.	*Zm PK1*	F-5'-AGATGTGAGAGTTGGTGATGAG-3'; R-5'-GGCACGTGGCAACAATAAA-3'
10.	*Zm AGPAse*	F-5'-AGACGAGATCTCAAGGCTACT-3'; R-5'-GACGCCCTCTTTGTTTGTTATG-3'
11.	*Zm PEPCase*	F-5'-AGCAGCTCAGGGACAAATAC-3'; R -5'-GGAATGGATCGCCTTCAAGA-3'
12.	*Zm OPAQUE*	F-5'-ATGATCCGTGGCTTACCAATTA-3'; R-5'-TTTCGAGAACTTCCCACATCC-3'
13.	*Zm MGDG2*	F-5'-AGAGGGTTCGAGACACAGAT-3'; R-5'-TCCTGTCCAGGGATGAAGT-3'
14.	*Zm DGDG1*	F-5'-GTGAGGACTCGCAAGAAGTT-3'; R-5'-CCTTGTACCCATGGAGTGAATTA-3'
15.	*Zm PIS*	F-5'-TGCTGATGAGGAGTCAACAAG -3'; R-5'-CAGCCAACTAGAGTGGAAACA-3'
16.	*Zm FAD*	F-5'-ATTTGTTGCTTGGCTGGATATG-3'; R-5'-GTCGCTAACATAGTGGTCTTGC-3'
17.	*Zm DGK1*	F-5'-GGGAACCATAGAGGTTTCTCATC-3'; R-5'-TCCAAGATGTCGGCCATTAC-3'
18.	*Zm PLC*	F-5'-ATGGAGGGACATTGACTGC-3'; R-5'-CGCTGCCACTGCGATTA-3'
19.	*Zm Bz2*	F-5'-CACGGCCTCTCACTCATC-3'; R-5'-TGAACTGCACCACCTTCTC-3'
20.	*ZmCHS*	F-5'-CGACTGGAACTCCATCTTCTG-3'; R-5'-TGGACATGTTGCCGTACTC-3'
21.	*Zm FLS*	F-5'-CTCCAGGTCTTCAAAGATGGTC-3'; R-5'-CATGACATCCGCGTCTTCTC-3'
22.	*Zm DHFR1*	F-5'-AGTTTGTCCCACGAGCTATG-3'; R-5'-GATGTGGACGTATCCCATTCTT-3'
23.	*Zm CAD1*	F-5'-CGGTGATGCTAACCAGATGAA-3'; R-5'-GCTTGAGGAGAGCAAGGTAAG-3'
24.	*Zm UBq1*	F-5'-CAGAGGTGGTATGCAGATCTTT-3'; R-5'-CTGGATCTTCGCCTTCACAT
25.	*Zm Tub*	F-5'-CACTGATGTTGCTGTCCTGC-3'; R-5'-CGCTGTTGGTGATTTCGG-3'

Supplementary Table 2: Total P content in the 0.01 % *Ascophyllum nodosum* extract

	**Total P content**
0.01 % ANE	5 µM
